# Editorial: School achievement and failure: Prevention and intervention strategies

**DOI:** 10.3389/fpsyg.2022.838057

**Published:** 2022-10-21

**Authors:** Edgar Galindo, Adelinda Araújo Candeias, Małgorzata Lipowska, Oscar Conceição de Sousa, Marcus Stueck

**Affiliations:** ^1^Comprehensive Health Research Center (CHRC), University of Evora, Évora, Portugal; ^2^University of Gdansk, Gdańsk, Poland; ^3^Universidade Lusófona de Humanidades e Tecnologias, Lisbon, Portugal; ^4^Academy of Work and Health (DPFA), Leipzig, Germany

**Keywords:** school achievement, school failure, academic achievement, academic self-efficacy, learning difficulties, mathematics achievement

School achievement is probably dependent on a set of social, psychological, family and environmental factors acting jointly in a complex way to foster learning (Christenson et al., 2001); more recent studies devoted to school refusal and academic achievement emphasize once again the complex relationship of individual and contextual factors in these matters (Filippello et al., 2019). At the same time, school failure seems to be a long process of disengagement from learning, often beginning in elementary school, in which the same set of factors plays a central role. So, attending school and classes, completing schoolwork, and participating in school activities are correlated with a positive school performance. Whereas, missing classes, not completing schoolwork, and getting low grades are correlated with school failure, as well as with problems like engaging in disruptive behavior, disinterest in school and low expectations of success. Educational agencies worldwide are increasingly concerned about school failure and, in turn, about the factors determining school achievement. Generally, school failure refers to children, adolescents or adults who have dropped out of school before acquiring basic education, or have completed basic education without going further, or who have dropped out of school before acquiring lower secondary education, or even those who have completed lower secondary education but dropped out before graduating at a higher level. The consequences of school failure seem to be severe and long lasting. These students are more likely to have mental health problems, to abuse of drugs, to commit crimes, to be unemployed, and to earn lower salaries (Lagana-Riordan et al., 2011).

In spite of constant efforts to solve the problem (see Eurydice, 1994; U.S. Department of Education, 2002), it persists. For instance, Carrie and Ballenger (2016) note that in the USA more than 1.3 million students drop out each year. Clycq et al. (2013) point out that in spite of the high performance level of students in Belgium, school achievement depends strongly on SES and ethnic background. In Latin America, the average number of students who are two or more years overage in primary school (an index of school delay) for the region is 10%, and the percentage of students who did not reach the minimum school level (sixth grade) varies between 51% in Cuba and 94% in Nicaragua (UNESCO, 2022). Developing effective approaches to reducing school failure in all the different definitions is consequently a high priority in education.

School achievement and failure seem to be the result of multiple social, political, and individual factors. The origins of school failure are complex and are not limited to school, because family and community risk factors can foster or inhibit the individual's cognitive, social and emotional development. Ethnic minorities, students from low socioeconomic backgrounds, students with disabilities, and students taught in a second language are risk groups.

In this context, psychologists all over the world are researching the factors determining school failure, looking for prevention and intervention strategies. Individually, school achievement depends on cognitive, emotional, and behavioral factors. Children need social, cognitive, and emotional skills starting in kindergarten in order to be academically successful. Critical factors like motivation, social control, self-regulation, self-representations, parental involvement, and attitudes toward school are important. Nevertheless, the study of determining factors is only the first step to finding sound and scientifically based prevention and intervention strategies.

The content of this book reflects the state of the art in the research on school achievement and failure in Europe, Asia, North, and South America. The study of intervention and prevention strategies is increasingly important, while the analysis of the causes of school achievement and failure is still attracting researcher attention. Consequently, four studies are devoted to intervention strategies, three to prevention issues, and five analyze some of the social, emotional or cognitive variables affecting school achievement.

In the field of intervention, Cvencek et al. conducted a Spanish study designed to boost children's mathematical self-concept. They applied a multicomponent intervention combining explicit and implicit approaches to helping third grade school children to form more positive beliefs about math and themselves. They conclude that self-concepts can be taught, and social-cognitive interventions can boost children's beliefs about themselves and produce better math achievement. Also in Spain, Bermejo et al.b successfully applied a constructivist intervention program for the improvement of mathematical performance in first- and second-graders. The program established students as the main agents of their learning with their teachers serving as guides. The mathematical content was organized in terms of the complexity and significance for the student and cooperative work strategies were implemented. The authors suggest constructivist instruction seems to have positive effects on the acquisition of mathematical skills. Tordön et al. implemented a strategy to improve school performance using individual assessments and school-based interventions in Swedish children from 8 to 15 years of age living in foster care. The results showed improved skills in complex aspects of literacy, mathematics, and cognitive performance. It is therefore concluded that higher-order cognitive functions can develop positively when appropriate school support is provided.

Concerning prevention strategies,Urbina-Garcia applied a program to facilitate the transition from preschool to first grade in Mexican primary schools. The program comprised activities to help students develop cognitive, social and motor skills, as well as to promote home-school links and preschool-school links. The positive results show the importance of the training of previous skills for school children's successful development. Baker-Henningham et al. implemented a violence-prevention program in Jamaican preschools and observed the later effects on first grade achievement. They found that promoting child social-emotional competence led to positive outcomes across multiple child developmental domains. López et al. explored the effects of psychologists and social workers working in the Chilean school system on the academic achievement of 8th- to 10th-grade students and school dropouts. They analyzed national records of non-teaching professionals working as school staff members, looking for correlations with achievement scores on national tests. They found that the presence and number of school psychologists per school seemed to be positively associated with higher math achievement.

In the analysis of causes, Borchet et al. explored in Poland to what extent diverse types of parentification relate to academic achievement in adolescents. They found a positive relation between instrumental parentification and school achievement. Also in Poland, Rózycka-Tran et al. studied the influence of parent-child relations on academic performance in college students, comparing two different cultures, one individualistic (Poland) and the other collectivistic (Vietnam). The results show that in a more individualistic culture, a reciprocal mode is stronger predictor of study engagement, whereas the authoritarian mode is stronger in a collectivistic culture. In Sweden, Bertills et al. carried out a longitudinal study to analyze the effect of cognitive variables on Physical Education (PE) achievement in secondary school students with and without disabilities. They investigated the development of participation constructs as perceived by students, in order to explore how children's PE self-efficacy perceptions, general school self-efficacy and aptitude for participating in PE changed over time, and how these changes determined achievement. Bermejo et al.a analyzed in Spain the effects of teaching in a language different from the students' mother tongue on the mathematical thinking of 7–8-year-old children. They conclude that students who were taught mathematics in a second language had similar skills for resolution of algorithms to those students whose teaching language coincided with their mother tongue, but they had more difficulties in the resolution of word problems or in the tests of mathematical competence. Assari et al. explored the factor parental education level on school performance of adolescents and young adults living in poor neighborhoods in the United States. They conclude that a high level of parental education is a salient determinant of educational outcomes, although to a lesser extent in less privileged neighborhoods. Finally, Kaloeti et al. analyzed in Indonesia a problem deeply related to school performance, namely peer bullying at school. They found that bullying significantly influences the onset of anxiety in 11–13-year-old-children, particularly cyberbullying.

We hope these studies will help to broaden and deepen the path toward finding sound, scientifically based prevention and intervention strategies to combat school failure.

## Author contributions

All authors listed have made a substantial, direct, and intellectual contribution to the work and approved it for publication.

## Funding

This work was carried out with support from the University of Evora (Portugal), the University of Gdansk (Poland), the Universidade Lusófona de Humanidades e Tecnologias (Portugal), and the Academy of Work and Health (Germany).

## Conflict of interest

The authors declare that the research was conducted in the absence of any commercial or financial relationships that could be construed as a potential conflict of interest.

## Publisher's note

All claims expressed in this article are solely those of the authors and do not necessarily represent those of their affiliated organizations, or those of the publisher, the editors and the reviewers. Any product that may be evaluated in this article, or claim that may be made by its manufacturer, is not guaranteed or endorsed by the publisher.
